# The Cognition and Affect after Stroke - a Prospective Evaluation of Risks (CASPER) study: rationale and design

**DOI:** 10.1186/s12883-016-0588-1

**Published:** 2016-05-12

**Authors:** Elles Douven, Syenna H. J. Schievink, Frans R. J. Verhey, Robert J. van Oostenbrugge, Pauline Aalten, Julie Staals, Sebastian Köhler

**Affiliations:** Alzheimer Center Limburg and School for Mental Health and Neuroscience (MHeNS), Department of Psychiatry and Neuropsychology, Maastricht University Medical Center (MUMC+), Maastricht, The Netherlands; Cardiovascular Research Institute Maastricht (CARIM), Department of Neurology, and School for Mental Health and Neuroscience, Maastricht University Medical Center (MUMC+), P.O. BOX 616 (DRT12), 6200 MD Maastricht, The Netherlands; Cardiovascular Research Institute Maastricht (CARIM), Department of Neurology, Maastricht University Medical Center (MUMC+), Maastricht, The Netherlands

**Keywords:** Stroke, Cognition, Dementia, Depression, Apathy, Neuroimaging, Design

## Abstract

**Background:**

Cognitive impairment and neuropsychiatric syndromes, like depression and apathy, are frequent residual consequences of stroke. These have a large impact on quality of life and long-term prognosis. Several factors are involved in the development of these residual syndromes, although their exact role and their interrelationships remain still rather unclear. The Cognition and Affect after Stroke: a Prospective Evaluation of Risks (CASPER) study has been primarily designed to examine whether stroke-specific (e.g. lesion location, volume, type, severity), cerebrovascular and neurodegenerative (e.g. white matter changes, atrophy, microbleeds, perivascular spaces), inflammatory, endothelial, and (epi)genetic markers are associated with cognitive impairment, post-stroke depression, and post-stroke apathy, and whether they predict their course over 12 months. The secondary aims are to investigate how the above-mentioned markers interact with each other, and to determine if patients with apathy and depression after stroke differ in pathogenesis, course, and outcome (e.g. functional outcome, neurocognitive performance, quality of life).

**Methods/design:**

CASPER is a 1-year prospective clinical cohort follow-up study in 250 stroke patients recruited at the neurological in- and outpatient services at Maastricht University Medical Center (MUMC+, Maastricht, The Netherlands), and Zuyderland Medical Center (Sittard and Heerlen, The Netherlands). At baseline (3 months post-stroke), a neuropsychological assessment, neuropsychiatric interview, blood sample, and brain magnetic resonance imaging (MRI) scan are conducted. Assessment of neuropsychiatric and neurocognitive status are repeated 6 and 12 months later.

**Discussion:**

The CASPER study investigates stroke-specific, vascular, neurodegenerative, inflammatory, and genetic markers of the development of vascular cognitive impairment, depression, and apathy after stroke. This creates the possibility to study not only the contribution of these individual markers but also their joint contribution, which differentiates this study from earlier stroke cohorts who lacked long-term follow-up data, a large sample size, an extensive MRI protocol, and markers from the blood. The knowledge we derive from this study might help in identifying markers that are associated with, or can predict the onset, maintenance, and progression of vascular cognitive impairment, depression, and apathy after stroke, and could provide new insights into possibilities for treatment and rehabilitation that result in better functional outcome after stroke.

**Trial registration:**

ClinicalTrials.gov NCT02585349

## Background

Stroke is one of the leading causes of disability in the Western world. According to the Global Burden of Disease Study in 2010 the stroke incidence worldwide was around 17 million persons per year, with 33 million people being still alive after stroke, with about 70 % of all stroke patients staying with residual symptoms [1]. Stroke is the second most frequent cause of death, after coronary artery disease [2]. As a result of the aging population, this number will probably increase steadily in the next decades [3]. At the same time, case fatality rates are declining due to better acute treatment. Therefore, more and more individuals will need to learn how to deal with the residual disabilities and handicaps [1]. Frequent impairments after stroke are cognitive impairments and neuropsychiatric syndromes, including depression and apathy [4, 5] which have an impact on long-term prognosis (higher mortality and more disability) and quality of life (QoL) of stroke survivors [6, 7].

### Vascular cognitive impairment

Vascular cognitive impairment (VCI) describes the full spectrum from mild to severe cognitive impairment in people with cerebrovascular disease, including vascular mild cognitive impairment and vascular (or post-stroke) dementia (VaD) [8]. Risk factors of VCI after stroke include event-related factors such as infarct severity, lesion volume, age, low education, history of diabetes or atrial fibrillation, and number of recurrent strokes [9]. The risk for post-stroke dementia is known to be highest within the first year, with an estimated incidence rate of 20–30%, which is nine-times the general population’s risk [9].

It has been suggested that both vascular and neurodegenerative pathologies contribute to post-stroke VCI [10]. Cognitive impairments can be the result of strategic infarcts, but (pre-existing) white matter hyperintensities (WMH) and atrophy of the medial temporal lobes can contribute too, and they probably interact with each other, though the nature of these interactions is not well understood [11]. In addition, a higher risk for VCI has been reported in carriers of the Apolipoprotein E (ApoE) ε4 allele, which is the major genetic risk factor for Alzheimer’s disease (AD) dementia [12]. Indeed, evidence accumulates that AD and VaD should not be regarded as mutually exclusive diagnoses, but rather as a continuum with pure AD and VaD at its extremes, and most people showing mixtures of both type of pathologies [13]. While the dominating view is that vascular changes work as a catalyst of primary neurodegenerative changes in AD it is largely unknown whether the opposite is true for VCI, i.e. whether AD-related changes work as a catalyst for post-stroke dementia.

Next to neurodegeneration, neuro- and vascular inflammation might contribute to VCI by partly mediating the pathophysiology underlying VCI as part of a final common pathway [14]. Following stroke, increased auto-immune activity is a common response, and it has been shown that up-regulated inflammation markers like C-reactive protein (CRP), as a consequence of a prolonged auto-immune response, relate to poor prognosis. This includes a higher risk of additional future strokes and mortality, but also more severe VCI [15, 16]. Other enzymes, molecules, and ligands (myeloperoxidase, soluble intercellular adhesion molecule, soluble vascular cell adhesion molecule-1, soluble E selectin, soluble P selectin and CD40 ligand) are involved in vascular inflammation as part of an inflammatory response to stroke, and have also been found to be increased in AD [17–20]. Also, up-regulation of proinflammatory cytokines (interleukin-6 and tumor necrosis factor alpha) and proteins (high sensitivity CRP) are risk factors for both dementia [21, 22] and cardiovascular disease [23]. It is unknown whether similar inflammation mechanisms are involved in cognitive deterioration in VCI, and therefore, research is needed to investigate whether inflammation contributes to VCI independently or in joint action with other (e.g. neurodegenerative) factors.

Finally, genetic factors are also suggested to play a role in the pathogenesis of VCI, but in contrast to AD, evidence is still scarce. A genome-wide association study in VaD identified a novel genetic locus near the androgyn receptor, and this finding was replicated in two independent validation datasets [24]. Polymorphisms and mutations on the genes coding for angiotensin-converting enzyme and methylenetetrahydrofolate reductase are risk factors for cardiovascular diseases and might be related to the development of VaD, although this relationship is still controversial [25]. For AD, several genetic risk loci are already identified next to ApoE, including clusterin (CLU), phosphatidylinositol binding clathrin assembly protein (PICALM), and encoding complement component [3b/4b] receptor 1 (CR1) [26], but their effects are generally small (see http://www.alzgene.org). Whether they contribute to post-stroke dementia is not known.

### Post-stroke depression

Depression is a common neuropsychiatric syndrome following stroke. According to a recent systematic review by Hackett et al. [27], around one-third of the stroke survivors experiences depression in the first 3 months post-stroke, and studies with long-term follow-up have shown that it is often a chronic disorder, with a remitting-relapsing pattern [28]. Patients with post-stroke depression (PSD) have worse functional recovery [29], a higher risk for cognitive impairment [7], and higher mortality risk [30, 31].

Early studies implied that PSD is mainly associated with anterior lesions in the left hemisphere, however, a major systematic review and meta-analysis found no support for this ‘lesions location hypothesis’ [32]. Vataja et al. [33] suggested that lesions in fronto-subcortical regions in general are involved in developing PSD, often accompanied by impairments in executive functioning, leading to what has been called the ‘depression-executive dysfunction syndrome’. Other factors that have been associated with the development of PSD are cognitive impairment, stroke severity, physicial disability, and pre-stroke depression and anxiety [7]. Diminished QoL and also low social support can both contribute independently to the severity of depression [34]. A study by van Mierlo et al. [35] investigated the association between a broad range of psychological factors and PSD with a multivariable logistic regression analysis. They showed that more passive coping and more helplessness, less acceptance and less perceived benefits were all significantly and independently associated with symptoms of post-stroke depression. Furthermore, fatigue after stroke has been associated with PSD, although not all patients with fatigue develop symptoms of depression and vice versa [28].

Neuroinflammation might also play a role in the underlying mechanisms of PSD. Levels of CRP, soluble E selectin, CD40 ligand, interleukin-6, tumor necrosis factor alpha, and high sensitivity CRP are deregulated in patients diagnosed with major depressive disorder [14, 18, 22, 23, 36–38]. In addition, high plasma levels of neopterin are found in people with depression, and might predict the development of PSD [39]. Markers of endothelial function might be important as well, since increased expression of soluble intercellular adhesion molecule and soluble vascular cell adhesion molecule-1 are associated with depression [40–43]. Although several inflammation markers have been related to symptoms of depression, studies examining these associations in PSD specifically are relatively scarce. Some studies found an elevation in interleukin-6 and tumor necrosis factor alpha in PSD [44]. Recently, a study by Tang et al. [39] showed an association between elevated serum levels of neopterin in the acute phase after stroke and PSD at 6-month follow-up.

High plasma levels of homocysteine are known to increase the risk for cerebrovascular disease, but have also been associated with depressive disorders. Increased levels of homocysteine result in cerebrovascular disease and a deficiency in monoamine neurotransmitters, which might lead to a depressed mood [45]. Homocysteine, folate and vitamin B12 are all involved in methylation reactions that are necessary for monoamine neurotransmitter production, but also the production of phospholipids and nucleotides. A deficiency in folate and vitamin B12 has also been associated with depressive disorders [46].

### Post-stroke apathy

Apathy has been defined as a disorder of diminished motivation, characterized by lowered initiative, restricted engagement in social interactions and activities, diminished cognitive activities, and lack of emotional response [47]. It was traditionally seen as a symptom of other syndromes (e.g. depression and dementia), but mounting evidence suggests that it might be an independent syndrome with a different etiology [48–50]. Post-stroke apathy (PSA) is as frequent as PSD, with a mean prevalence rate of 34.6 % at 4 months post-stroke [5]. However, PSA gained relatively less attention in research in comparison with PSD, and most research focuses on the difference between the two.

While PSD is suggested to be associated with left anterior lesions [51, 52], PSA has been associated with right hemispheric subcortical lesions, particularly in the basal ganglia and in the anterior cingulate circuit which is involved in motivational processes [53–55]. Other factors associated with PSA are older age, lower education, and severity of VCI [4, 5, 51, 56]. Furthermore, both PSD and PSA have been associated with poor functional recovery and low QoL [5, 29]. Overall, lack of longitudinal studies, differences between time of measurement after stroke (acute phase/chronic phase), and lack of studies with a sufficient sample size make it difficult to interpret study results.

In conclusion, several factors play a role in the development of VCI, PSD, and PSA. However, most studies investigated the underlying mechanisms in isolation and did not take into account how several factors interact with each other. The Cognition and Affect after Stroke, a Prospective Evaluation of Risks (CASPER) study incorporates a broad range of psychosocial, blood and neuroimaging markers to be able to study their role alone and in combination with each other to predict individual differences in the onset and course of the cognitive and neuropsychiatric consequences of stroke.

### Study aims

The primary aim of CASPER is to identify stroke-related, cerebrovascular, neurodegenerative, (epi)genetic, endothelial and inflammation markers that are associated with VCI, PSD, and PSA in patients with ischemic or hemorrhagic stroke. The secondary aims are to investigate how the above-mentioned markers interact with each other, and to determine if patients with apathy and depression after stroke differ in pathogenesis, course, and outcome (e.g. functional outcome, neurocognitive performance, quality of life).

The main research questions are:

1. Are stroke-specific, additional vascular, neurodegenerative, inflammatory or genetic markers a) associated with VCI at 3 months after stroke and b) predictive for its course over 12 months?
2. Are stroke-specific, additional vascular, neurodegenerative, inflammatory or genetic markers a) associated with PSD and PSA at 3 months after stroke and b) predictive for their course over 12 months?
3. Do the above-mentioned markers interact on VCI, PSD, and PSA outcome?
4. Which psychosocial factors are associated with the development of VCI, PSD, and PSA?
5. Do PSA and PSD differ in their pathogenesis, cognitive profile, course, and outcome?

## Methods

### Study design

CASPER is a prospective clinical cohort study into cognitive impairments and neuropsychiatric syndromes after stroke with a follow-up of 1 year. Baseline measurements take place in the post-acute phase after stroke (i.e. 10 to 12 weeks post-stroke) to assess presence and severity of VCI, PSD, and PSA. Serial assessments take place at 6 and 12 months after baseline. The total duration of the study will be approximately 3.5 years, from June 2013 (first in) until November 2016 (last out).

### Patients

In total, 250 consecutive patients with either ischemic or hemorrhagic stroke are included. Patients who are admitted to the Stroke Unit of the Maastricht University Medical Center + (MUMC+), Maastricht, the Netherlands, the Stroke Unit of Zuyderland Medical Center in Sittard-Geleen and Heerlen, the Netherlands, or those who visit the Transient Ischemic Attack clinic of MUMC+ for a non-fatal ischemic or intracerebral hemorrhagic stroke and fulfill the in- and exclusion criteria are asked to participate. Stroke is defined as a clinical stroke syndrome (sudden neurological dysfunction lasting >24 h, with no apparent cause other than that of vascular origin). The stroke can be a first-ever event or a recurrent supra- or infratentorial stroke in a patient who recovered from a previous event without obvious residual symptoms. Ischemic strokes might be cortical or lacunar, and could be included with or without evidence of ischemia on clinical brain scan. Hemorrhagic strokes are non-traumatic deep, lobar, cerebellar, or brainstem hemorrhages as evidenced by a clinical brain scan. Eligibility criteria are chosen to make sure that the sample will represent the current clinical situation (Table 1). Participation of an informant who knows the patient well enough to answer questions about his/her functioning over the last ± 10 years is desirable but not required.

**Table 1:** In- and exclusion criteria

Inclusion criteria	Exclusion criteria
- Ischemic or hemorrhagic stroke	- Subarachnoidal hemorrhage, traumatic hemorrhage, primary intraventricular hemorrhage and transient ischemic attack
- MMSE score ≥ 15 (to ensure valid testing)	- Age younger than 40 years (to exclude atypical strokes)
- Written informed consent	- Severe aphasia (as it interferes with performance on and understanding of the instructions of the neuropsychological tests)
- Sufficient knowledge of the Dutch language	- Evidence for pre-stroke dementia (based on clinical diagnosis or IQ-CODE) in the 5 years prior to the stroke
	- Other existing psychiatric and neurological diagnoses that are known to affect cognition (Parkinson’s disease, bipolar disorder, epilepsy, schizophrenia, or substance abuse)^a^

^a^Lifetime history of depression is not considered as a reason for exclusion as it might be a risk factor for post-stroke depression

### Assessments

The baseline measurement (T0) is scheduled 3 months post-stroke to avoid confounding effects by the acute event, including acute inflammatory responses. At T0, patients undergo a venipuncture, brain magnetic resonance imaging (MRI) scan, neuropsychological assessment and neuropsychiatric interview. They fill in several questionnaires to assess the presence and severity of PSD and PSA, and to evaluate other factors as functional ability, fatigue, personality, and QoL. A semi-structured interview with an informant is performed to provide additional information about functional outcome of the patient and presence of neuropsychiatric syndromes. Six (T1) and 12 months (T2) after T0, the neuropsychological assessment and questionnaires are repeated. The venipuncture, MRI scan, neuropsychological assessment, and neuropsychiatric interview all take place in the academic hospital in Maastricht and are performed by specially trained nurses (venipuncture), radiology assistants (MRI scan) and trained research (neuro)psychologists (neuropsychological assessment and neuropsychiatric interview). If a patient is not able to visit the hospital due to reduced mobility, the patients are visited at their current place of residence.

Patients who suffer from a recurrent stroke during the 12-month follow-up period will be followed-up normally, if the health status of the patient allows this, and if the patient does not suffer from severe aphasia after the recurrent stroke. Information about the type, location, and severity of the lesion of the recurrent stroke is collected, which also allows us to study the potential impact of recurrent strokes on our outcome measures. Recurrent strokes are taken into account in our analyses with longitudinal data to avoid confounding effects.

### Clinical data

In a standardized case record form, data on demographics (e.g. age, gender, educational level, ethnicity, marital status), medical history (e.g. personal and family history of cardiovascular, neurological or psychiatric disorders), medication use, physical examination (e.g. weight, height, blood pressure, cholesterol), and lifestyle (smoking behavior, alcohol consumption, drug use, physical activity) are collected.

### Neuropsychological assessment and cognitive endpoints

The neuropsychological assessment consists of a standardized battery of cognitive tests measuring specific cognitive domains: global cognition, episodic memory, working memory, information processing speed, executive functioning, visuoconstruction, neglect, premorbid IQ, and language (dysphasia). An overview of all test instruments and their cognitive domains is presented in Table 2. The neuropsychological tests are administered according to a standardized test protocol by trained research (neuro)psychologists.

**Table 2:** Neuropsychological measures and neuropsychiatric questionnaires

	Instrument	T0^a^	T1^a^	T2^a^
Global cognition	MMSE [89]	X	X	X
Episodic Memory	15-Word Verbal Learning Test [90]	X	X	X
Working memory	Digit-span of the WAIS-III [91]	X	X	X
Information processing speed	Digit Symbol Substitution Test (DSST) Trail Making Test (TMT; part A and B) [91, 92]	X	X	X
Executive functioning	TMT (interference), BADS Zoo Map & Key Search, Verbal Fluency (60 s; animals and professions) [92–94]	X	X	X
Visuo-construction	Clock drawing [94]	X	X	X
Neglect	Star cancellation [95]	X		
Premorbid IQ	NLV [96]	X		
Language/aphasia	Boston Naming Test [97]	X		
Depression	MINI, MADRS, HADS, NPI [62–64, 68]	X	X	X
Apathy	AES, NPI [66, 68]	X	X	X
Premorbid dementia	IQ CODE [60]	X		
Functional ability	Barthel Index, Lawton [73, 74]	X	X	X
Quality of life	SS-QoL [75]	X	X	X
Fatigue^15^	FSS [72]	X	X	X
Personality^16^	NEO-FFI [76]	X		

^a^T0: baseline measurement, T1: 6-month follow-up, T2: 12-month follow-up

*Abbreviations*: *MMSE* Mini Mental State Examination, *WAIS* Wechsler Adult Intelligence Scale, *BADS* Behavioral Assessment of the Dysexecutive Syndrome, *NLV* Nederlandse leestest voor volwassenen (Dutch version of the National Adult Reading Test), *MINI* Mini International Neuropsychiatric Interview, *MADRS* Montgomery-Åsberg Depression Rating Scale, *HADS* Hospital Anxiety and Depression Scale, *NPI* Neuropsychiatric Inventory, *AES* Apathy Evaluation Scale, *IQ-CODE* Informant-Questionnaire on Cognitive Decline in the Elderly, *SS-Qol* Stroke Specific Quality of Life, *FSS* Fatigue Severity Scale, *NEO-FFI* NEO Five Factory Inventory

VCI is defined as a score ≤ 1.5 standard deviations below the general population mean in one or more cognitive domains, based on available norm scores for age, gender, and level of education for the Dutch general population. In addition, there should not be any interference in daily activities and no diagnosis of dementia according to DSM-V criteria [57]. The impairments have to represent a significant decline from premorbid levels of functioning. The diagnosis and type of dementia is made by an experienced neuropsychiatrist or neuropsychologist based on DSM-V criteria [57]. A consensus meeting is arranged when there is a discrepancy in diagnosis, and if no consensus can be reached, the patient will be considered not demented. The diagnosis of AD is made according to the standardized clinical criteria for AD based on the National Institute for Neurological and Communicative Disorders and Stroke-Alzheimers Disease and Related Disorders Association (NINCDS-ADRDA) criteria [58]. The diagnosis of VaD is made according to the criteria proposed by the National Institute of Neurological Disorders and Strokes – Association Internationale pour la Recherche et l’Enseignement en Neurosciences (NINDS-AIREN) [59].

The Informant Questionnaire on Cognitive Decline in the Elderly (IQ-CODE; short form) is used to detect possible dementia prior to the index stroke, and is rated retrospectively (past 5 years prior to the index stroke) [60], in combination with information from medical records. A cut-off score ≥ 3.60 is used to indicate possible dementia prior to the stroke, as has been used in a previous study with stroke patients [61].

### Neuropsychiatric assessment

#### Depression

The symptoms of a major depressive disorder according to DSM-IV and V are assessed with the Mini International Neuropsychiatric Interview (M.I.N.I.) [62]. The MINI is a semi-structured interview administered to the patient and consists of three different parts, which aim to determine whether the patient can be diagnosed with a current major depressive disorder, a depressive episode lifetime, and a current dysthymic disorder. The clinician-rated Montgomery-Åsberg Depression Rating Scale (MADRS) is used to assess the severity of symptoms of depression [63]. This rating scale consists of 10 items to evaluate the severity of depressive symptoms and is filled out by the clinician [63]. In addition, the Hospital Anxiety and Depression Scale (HADS), a 14-item self-report scale, is used to identify clinically significant levels of anxiety and depression [64]. According to a study by Kang et al. [65], the HADS and MADRS are especially valid as screening instrument for PSD (in both the acute and chronic phase), since these scales focus less on the presence of somatic symptoms, resulting in less misclassification.

#### Apathy

The Apathy Evaluation Scale (AES) is an 18-item scale used to evaluate the presence and severity of apathy [66]. Three different versions of the AES are available, a clinician-rated (administered to the patient), informant-rated, and a patient-rated version. The AES has been rated as the most favorable instrument for assessing apathy, with the clinician-rated version being most valid of the three different versions [67]. Both the informant-rated and clinician-rated version (administered to the patient) of the AES are used in the CASPER study.

#### Generic

The Neuropsychiatric Inventory (NPI) is administered as a semi-structured interview to the informant of the patient [68]. The NPI is originally developed to evaluate neuropsychiatric symptoms in dementia, but is also frequently used in the stroke population [28]. It evaluates the presence and severity of 12 different neuropsychiatric symptoms (delusions, hallucinations, agitation, depression, anxiety, euphoria, apathy, disinhibition, irritability, aberrant motor behavior, sleep, and appetite) [69]. The validity and reliability of the NPI, including the Dutch version, has been well established [70, 71].

### Additional interviews

Fatigue after stroke is assessed with the Fatigue Severity Scale (FSS) [72]. This 9-item self-rating scale can be answered on a 7-point scale, with higher scores representing more fatigue. The Barthel Index [73] is used to evaluate impairments in activities of daily living (ADL), and the Lawton Scale [74] is used to assess impairment in instrumental ADL. Furthermore, the Stroke Specific Quality of Life Scale (SS-QoL) is used to evaluate health-related QoL, which is defined as the physical, psychological, and social aspects of life that can be influenced by a change in health status [75]. The NEO Five Factor Inventory (NEO-FFI) is used to assess the big-five dimensions of personality, which are extraversion, neuroticism, openness to new experiences, agreeableness, and conscientiousness [76].

### Neuroimaging

A standardized brain MRI protocol is used as described in Table 3. The MRI scans are all performed on the same 3.0 Tesla head-only scanner equipped with an 8-channel head coil (Philips Achieva, Philips Medical Systems, Best, The Netherlands), using the same standardized scanning protocol for every patient. This protocol has been optimized for stroke and WMH segmentation, as well as segmentation of the hippocampus [77]. One scanning session takes approximately 30 min. After acquisition of the MR images, an anonymisation procedure is performed, to make sure that all imaging data are pseudonymised.

**Table 3:** Brain MRI protocol

Sequence name/Acquisition method	Field of View (mm)	Matrix	Slices	Inter-slice gap (mm)	Thickness (mm)	Voxel (mm)	TR /TE/TI (ms)
Coronal 3D T1-weighted (TFE)	240 × 240	240 × 240	160	0	1	1 × 1 × 1	8.2/3.8
2D Axial T2-weigthed (FSE)	230 × 185	328 × 225	48	0	3	0.7 × 0.8 × 3	3000/80
3D fluid-attenuation inversion recovery	250 × 250	228 × 226	283	0	1.1	0.6 × 1.1 × 1.1	8000/332/2400
T2* diffusion weighted imaging Gradient echo EPI	240 × 240	128 × 128	28	0	5	1.85 × 1.85 × 5	10000/72/2400
SWI	220 × 180	220 × 180	260	0	0.5	1 × 1 × 0.5	16/22

*Abbreviations*: *TR* repetition time, *TE* echo time, *TI* inversion time, *TFE* turbo field echo, *FSE* fast spin echo, *EPI* echo planar imaging, *SWI* susceptibility weighted imaging

### MRI markers

Several stroke characteristics are visually scored by an experienced vascular neurologist and consist of: presence and type of symptomatic stroke lesion (ischemic/hemorrhage), location of the lesion, number, type, and location of old infarcts and old hemorrhages, number and location of microbleeds, and degree of perivascular spaces in basal ganglia and centrum semiovale. The Fazekas visual rating scale is used to score deep and periventricular WMH [78]. In addition, a semi-automatic brain tissue segmentation program, based on the program used in the Rotterdam scan study [79], is used to measure WMH volumes and to calculate the volume of global brain atrophy, corrected for total intracranial volume. The accuracy of this semi-automated method appeared to be within the range of the inter-observer variability of fully manual segmentations but is less time-consuming while still requiring some manual correcting. The medial temporal lobe atrophy (MTA) visual rating scale [80] is used to indicate the level of atrophy in the medial temporal lobes (ranging from 0 to 3), separately for the left and the right hemisphere. Furthermore, hippocampal volume is measured based on atlas registration with high accuracy using the robust and validated method of learning embeddings for atlas propagation (LEAP) [81, 82].

### Blood markers

At baseline, fasting blood samples are taken. All patients take their morning medication after the venipuncture. In total, 35.5 mL of blood are taken and directly analyzed or stored. We measure several inflammation markers. In addition, standard lab procedures to assess levels of glucose, cholesterol (total, high-density lipoprotein cholesterol, low-density lipoprotein cholesterol), and triglycerides will be performed directly after the venipuncture [83–85]. Biomaterial (including deoxyribonucleic acid and ribonucleic acid) is stored anonymously for 15 years after the end of the study. Table 4 lists the blood markers investigated in CASPER.

**Table 4:** Blood sampling procedure

Tubes	Blood markers	Procedure
EDTA tube (4 × 6.0 mL)	Myeloperoxidase, neopterin, soluble intercellular adhesion molecule, soluble vascular cell adhesion molecule-1, soluble E selectin, soluble P selectin, CD40 ligand, interleukin-6, tumor necrosis factor alpha, Homocysteine	Transported on ice to the Biobank Maastricht UMC+ where the blood is preprocessed before storing. EDTA plasma is centrifuged and stored at −80 °C until analysis.
Natriumfluoride/EDTA tube (1x 4.0 mL) Coagulation tube (1 × 5.0 mL)	Glucose, total cholesterol high-density lipoprotein-cholesterol, low-density lipoprotein-cholesterol, triglyceride, folate, vitamine B12, high sensitivity C-reactive protein	Direct analyses of these markers are performed at the laboratory on the day of sample collection after centrifuging.
Buffycoats from the 4 EDTA tubes for DNA extraction	Apoliprotein E, clusterin, phosphatidylinositol binding clathrin assembly protein, encoding complement [3b/4b] receptor 1, angiotensin-converting enzyme,methylenetetrahydrofolate reductase	Buffycoats are taken from four EDTA tubes and separately stored at the Biobank until analysis.
Epigenetics 1 PAXgene tube (2.5 mL)	RNA	Blood is collected in PAXgene tubes to ensure long-term stability of ribonucleic acid and the tubes are stored at the Biobank. RNA extraction and subsequent analysis of expression of inflammatory and stroke-related genes at the mRNA level will be done.

*Abbreviations*: *mL* milliliter, *EDTA* ethylenediaminetetraacetic acid, *DNA* deoxyribonucleic acid, *RNA* ribonucleic acid, *mRNA* messenger ribonucleic acid

### Ethics, consent, and permissions

The study protocol has been approved by the Medical Ethics Committee of MUMC+. The research is performed according to the principles of the Declaration of Helsinki 59^th^ WMA General Assembly, Seoul, (October 2008) and in accordance with the Dutch Medical Research Involving Human Subjects Act (WMO). All eligible patients are informed about the aim of the study and both the patient and informant receive an information letter. All patients receive enough time to consider participation and if they agree to participate, an appointment is scheduled to sign informed consent. There are no risks for the patients participating in this study. All assessments in the study consist of routine operations and all patient data are pseudonymised. Contraindications for MRI are checked for every patient, and patients with contraindications are excluded for MRI. If a patient decides to withdraw from the study, this has no consequences or risks for the patients. Patients who refuse follow-up assessment are contacted to determine the reason for refusal.

### Power calculation and sample size

Based on results of the CODAS study [86], a previous study with stroke patients of our research group, it is expected that 50 % of the stroke patients will have VCI and 10 % will have VaD after 1 year. Furthermore, it is expected that 35 % of the patients will have minor or major depression after 1 year post-stroke [87]. A power calculation was performed with the freeware program G*Power version 3.1 (http://www.gpower.hhu.de) assuming categorical independent variables to which 50 % are exposed and 50 % are not exposed (median-split), since no reliable estimates are available for our main predictors from literature (MRI, inflammation, and genetics). Based on a 1-year follow-up, a two-sided alpha level of .05, and an expected drop-out rate of 20 % based on the CODAS study (about 10 % due to death), a sample size of 250 resulted in a power of 86 % to detect a relative risk of 2.00, or 80 % to detect a relative risk of 1.89 for PSD, and a 96 % power to detect a relative risk of 2.00, or 80 % power to detect a relative risk of 1.70 for VCI.

### Statistical analyses

Categorical data will be presented on group level expressed in absolute numbers and percentages, whereas continuous data will be presented by their mean, confidence interval, and standard deviation. In case of non-normality, continuous data will be presented as medians with their corresponding inter-quartile range. Differences between groups will be analyzed using t-tests for continuous variables or appropriate non-parametric counterparts, and chi-square tests for categorical variables. Pearson’s correlation coefficient will be used to assess the association between two continuous variables. Random effects models will be used to measure cognitive decline from baseline to follow-up, which has the advantage over repeated measures ANOVA that it is not restricted to study completers. The effect of baseline predictors on PSD, PSA, and VCI will be estimated with Poisson and Cox regression, taking into account differences in individual follow-up time. Furthermore, multivariate finite mixture modeling will be used to test the existence of separate PSD and PSA subtypes, using lesion location, WMH, inflammation markers, genetic risk factors, functional impairment, and cognitive functioning as subtype indicators. Age, gender, and education will be included as covariates in the multivariate models. Based on theory and statistical evaluation to see whether a variable might be a probable confounder it will be decided which additional covariates will be added on each analysis.

## Discussion

The CASPER study is a prospective clinical cohort study examining predictors of onset and course of VCI, PSD, and PSA using a deep phenotyping approach. It is an observational study with minimal risks for the participating patients. The longitudinal design creates the possibility to study independent predictors of VCI, PSD, and PSA. The CASPER study provides the possibility to study the course of these conditions and potential bidirectional relationships between them. A relatively large sample size and a MRI protocol that uses state-of-the-art volumetry of WMH and hippocampal atrophy make it more likely to find subtle differences in the brain’s macrostructure. Therefore, the CASPER study has an additional value to earlier stroke cohort studies (e.g. Sydney Stroke Study, The Fogarty-Mexico Stroke Cohort, CODAS) [10, 86, 88]. Furthermore, CASPER includes extensive blood-based biomarker data which results in a rich phenotyping approach generating both blood-based biomarker data and clinical brain MRI data, creating the possibility to study cerebrovascular, neurodegenerative, inflammatory, and genetic markers, and their inter-relationships. This is valuable since this gives a better account of how these changes occur in patients, namely in combination. Hence, the combination of these markers may predict individual differences in the development of VCI, PSD, and PSA better than each marker in isolation. Taken together, these methodological strengths promise to yield novel insights into the underlying mechanisms of the burdensome consequences of stroke.

However, some limitations of the study protocol have to be acknowledged here. Although the extensive in- and exclusion criteria were formulated with caution, they can possibly result in a less representative group of patients compared to the general stroke population. For instance, patients who are aphasic or who have pre-existent cognitive disturbances are excluded. No data are collected in the acute phase after stroke, which could yield additional information on the association between VCI, PSD, PSA and the earlier mentioned investigated markers, but a post-acute baseline time point is chosen to avoid confounding effects in the acute phase (e.g. generic acute inflammatory response, sickness behavior). MRI scans and venipuncture are only performed on T0, since it is not feasible due to logistical and financial reasons to repeat them at T1 and T2. However, if these data could also have been collected more information on the causes of VCI, PSD, and PSA could have been gathered, e.g. by linking longitudinal changes on MRI to changes in outcomes. Furthermore, patients with severe stroke are less probable to participate in the study, as the assessment is quite extensive. This can result in a cohort of patients with relatively mild stroke, which may influence the frequency of VCI, PSD, and PSA. Both ischemic and hemorrhagic strokes are included to increase the inclusion rate, however, due to the different etiologies of these strokes, it is likely that this limits the generalizability of the results. Therefore, we have to correct for stroke type in our analyses to take this into account. It is also likely that patients with depression or apathy after stroke are less motivated to participate in the study, resulting in an underestimation of the PSD and PSA prevalence in the study sample. In addition, the current follow-up period of 1 year allows testing predictions in the post-acute to early chronic phase, but is too short to study long-term consequences. Finally, CASPER allows for in-depth phenotyping and also covers a broad spectrum of factors impacting on the consequences of stroke, but it is not powered to test for more subtle associations or to use complex genetic models or testing of rare variants. Therefore, the study is also designed as to allow harmonization with other stroke cohorts in larger meta-studies as part of the STROKOG consortium (https://cheba.unsw.edu.au/group/strokog).

In conclusion, the CASPER study allows to study the role of several markers, consisting of cerebrovascular, neurodegenerative, and inflammatory markers, and their inter-relationships, in the development of VCI, PSD, and PSA. Furthermore, the longitudinal design and sample size provide the possibility to study the course of PSA, PSD, and VCI and to explore individual differences in their development. Better understanding of the role of all of these markers will make it possible to provide new treatment techniques or adapt existing treatment techniques, both on a biological and psychological level, which can result in better QoL and functional outcome of stroke patients.

### Ethics approval and consent to participate

The Medical Ethics Committee of MUMC+ approved the study. The research is performed according to the principles of the Declaration of Helsinki 59^th^ WMA General Assembly, Seoul, (October 2008) and in accordance with the Dutch Medical Research Involving Human Subjects Act (WMO). Participants will sign informed consent before participation.

### Consent for publication

Not applicable.

### Availability of data and material

No data were used for this manuscript. For inquiries or opportunities for working with CASPER data, please contact the corresponding author.

## Abbreviations

AD: Alzheimer’s disease
ADL: activities of daily living
AES: Apathy Evaluation Scale
ApoE: Apolipoprotein
ANOVA: analysis of variance
CASPER: Cognition and Affect after Stroke a Prospective Evaluation of Risks
CLU: clusterin
CODAS: COgnitive Disorders After Stroke
CR1: encoding complement component [3b/4b] receptor 1
CRP: C-reactive protein
DSM: Diagnostic and Statistical Manual of Mental Disorders
FSS: Fatigue Severity Scale
HADS: Hospital Anxiety and Depression Scale
IQ-CODE: Informant Questionnaire on Cognitive Decline in the Elderly
LEAP: learning embeddings for atlas propagation
MADRS: Montgomery-Åsberg Depression Rating Scale
MINI: Mini International Neuropsychiatric Interview
MRI: magnetic resonance imaging
MTA: medial temporal lobe atrophy
MUMC+: Maastricht University Medical Center
NEO-FFI: NEO Five Factor Inventory
NINCDS-ADRDA: National Institute for Neurological and Communicative Disorders and Stroke-Alzheimers Disease and Related Disorders Association
NINDS-AIREN: National Institute of Neurological Disorders and Strokes – Association Internationale pour la Recherche et l’Enseignement en Neurosciences
NPI: Neuropsychiatric Inventory
PICALM: phosphatidylinositol binding clathrin assembly protein
PSA: post-stroke apathy
PSD: post-stroke depression
QoL: quality of life
SS-QoL: Stroke Specific Quality of Life
VCI: vascular cognitive impairment
VaD: vascular dementia
WMH: white matter hyperintensities
WMO: Dutch Medical Research Involving Human Subjects Act
